# The influencing pathway of agrivoltaics on soybean protein concentration

**DOI:** 10.3389/fpls.2025.1618027

**Published:** 2025-09-02

**Authors:** Yuru Hu, Xueyan Zhang, Xin Ma

**Affiliations:** ^1^ Key Laboratory of Land Surface Pattern and Simulation, Institute of Geographic Sciences and Natural Resources Research, Chinese Academy of Sciences, Beijing, China; ^2^ Institute of Environment and Sustainable Development for Agriculture, Chinese Academy of Agricultural Sciences, Beijing, China; ^3^ Huanggang Meteorological Service, Huanggang, China

**Keywords:** agrivoltaics, soybean protein, critical pathways, nitrogen, logistic model

## Abstract

Agrivoltaics has the potential to enable simultaneous energy and food production. However, its impact on crop quality and the underlying mechanisms remain insufficiently understood, which poses a significant challenge to the development of agrivoltaic systems. This study aims to investigate the process and pathways through which agrivoltaic systems influence soybean protein concentration by examining crop responses to three types of photovoltaic structures: traditional photovoltaic panels, checkerboard photovoltaic panels, and translucent photovoltaic panels. The results indicate that: 1) soybeans grown under translucent photovoltaic panels exhibited no significant decrease in nitrogen accumulation or crude protein concentration compared to the control group; 2) logistic model analysis revealed that translucent photovoltaic panels outperformed traditional panels in terms of maximum nitrogen accumulation potential and the timing of peak accumulation, highlighting their relative advantage in preserving soybean protein concentration; 3) the effect of agrivoltaics on grain protein concentration is multifaceted, primarily involving nitrogen accumulation in leaves prior to the grain-filling stage and nitrogen translocation during the grain-filling stage. These findings provide robust empirical evidence and a theoretical framework for understanding how photovoltaic systems affect soybean quality and for developing strategies to mitigate any decline in quality. This research contributes to the future promotion and adoption of agrivoltaic systems.

## 1 Introduction

Agrivoltaics has been shown to effectively utilize solar radiation for both energy generation and food production (Dupraz et al., 2011), enhance agricultural economic efficiency (Choi et al., 2023), and support low-carbon agricultural practices (Pascaris et al., 2021; Ravi et al., 2016). The development of innovative technologies holds the potential to reduce photovoltaic installation costs, thereby improving the feasibility of agrivoltaic systems (China Meteorological Administration, 2023). By 2023, China’s installed agrivoltaic capacity had exceeded 40 GW, with more than 500 grid-connected projects completed (Xu, 2023). The widespread integration of field-grown crops is expected to play a crucial role in advancing agricultural emission reductions and climate change adaptation through agrivoltaics.

Soybean, a shade-tolerant and economically significant oil crop, has emerged as a promising candidate for agrivoltaic applications (Walston et al., 2022). Existing studies have demonstrated that integrating soybean cultivation with agrivoltaics can lower photovoltaic module temperatures, thereby enhancing photovoltaic conversion efficiency (Williams et al., 2023), as well as increasing land use efficiency and economic returns (Cuppari et al., 2021). Kim et al. (2021) explored the optimal combination of photovoltaics and soybean cultivation, with recent findings indicating that a shading level of up to 23% maximizes both energy output and farm income (Kim and Kim, 2023). While research has primarily focused on the impact of photovoltaic installations on soybean yield, prevailing evidence suggests that agrivoltaics may lead to yield reductions. For instance, Lee et al. (2022) reported an 18% decrease in soybean yield under a 28% shading rate due to an increase in seedless pods. Similarly, large-scale commercial agrivoltaic systems have shown an average 8% reduction in yield, accompanied by fewer pods (Potenza et al., 2022a). However, there remains a limited number of studies examining the effects of photovoltaic installations on soybean quality. Hu et al. (2024) proposed that photovoltaic modules may reduce levels of crude fat, soluble sugar, and starch in soybeans. Notably, the influence of agrivoltaic systems on soybean protein concentration an essential quality indicator has not yet been thoroughly investigated. Soybeans containing more than 42% protein are classified as high-quality. Meanwhile, photovoltaic installations reduce photosynthetically active radiation (PAR), potentially affecting carbon cycling. Yet, the mechanisms underlying nitrogen metabolism and their specific impacts on protein biosynthesis remain unclear and warrant systematic investigation.

The plant growth curve serves as a fundamental method for describing crop dry matter accumulation. Archontoulis and Miguez (2015) introduced a nonlinear model with improved horizontal asymptotes and fewer parameters for modeling the S-shaped growth curve. Commonly used models include the Richards, Gompertz, and Logistic models. The Richards model is particularly suitable for dense crop populations, where density significantly influences parameter estimation (Damgaard et al., 2002). Both the Gompertz and Logistic models are widely applied for estimating plant growth (Paine et al., 2012), with the Logistic model offering superior accuracy in representing growth dynamics (Jane et al., 2020). Yao et al. (2011) proposed a logistic curve specifically tailored for modeling nitrogen accumulation in soybeans. Moustakas et al. (2011) further refined this approach by dividing the growth stage into three phases, enabling precise description of nitrogen accumulation using the logistic equation. The Logistic model has been extensively employed in analyzing optimal farmland management strategies (Wang et al., 2019; Jahangirlou et al., 2023) and predicting yields (Wang et al., 2022), providing a reliable framework for assessing crop nitrogen uptake under agrivoltaic conditions.

In this study, a logistic model was employed to simulate the dynamic nitrogen accumulation process in agrivoltaic systems. This research aimed to address the current knowledge gap regarding the impact of photovoltaic modules distinguished by material, structure, and positioning on soybean nitrogen concentration and accumulation. Additionally, a modeling approach was developed to investigate the nitrogen absorption dynamics during soybean kernel development, identifying key parameters and pathways through which photovoltaic modules influence grain nitrogen accumulation. This model offers a scientific basis for optimizing photovoltaic module configurations and mitigating their impact on soybean nutrient uptake.

## 2 Materials and methods

### 2.1 Photovoltaic device design

**Figure 1:**
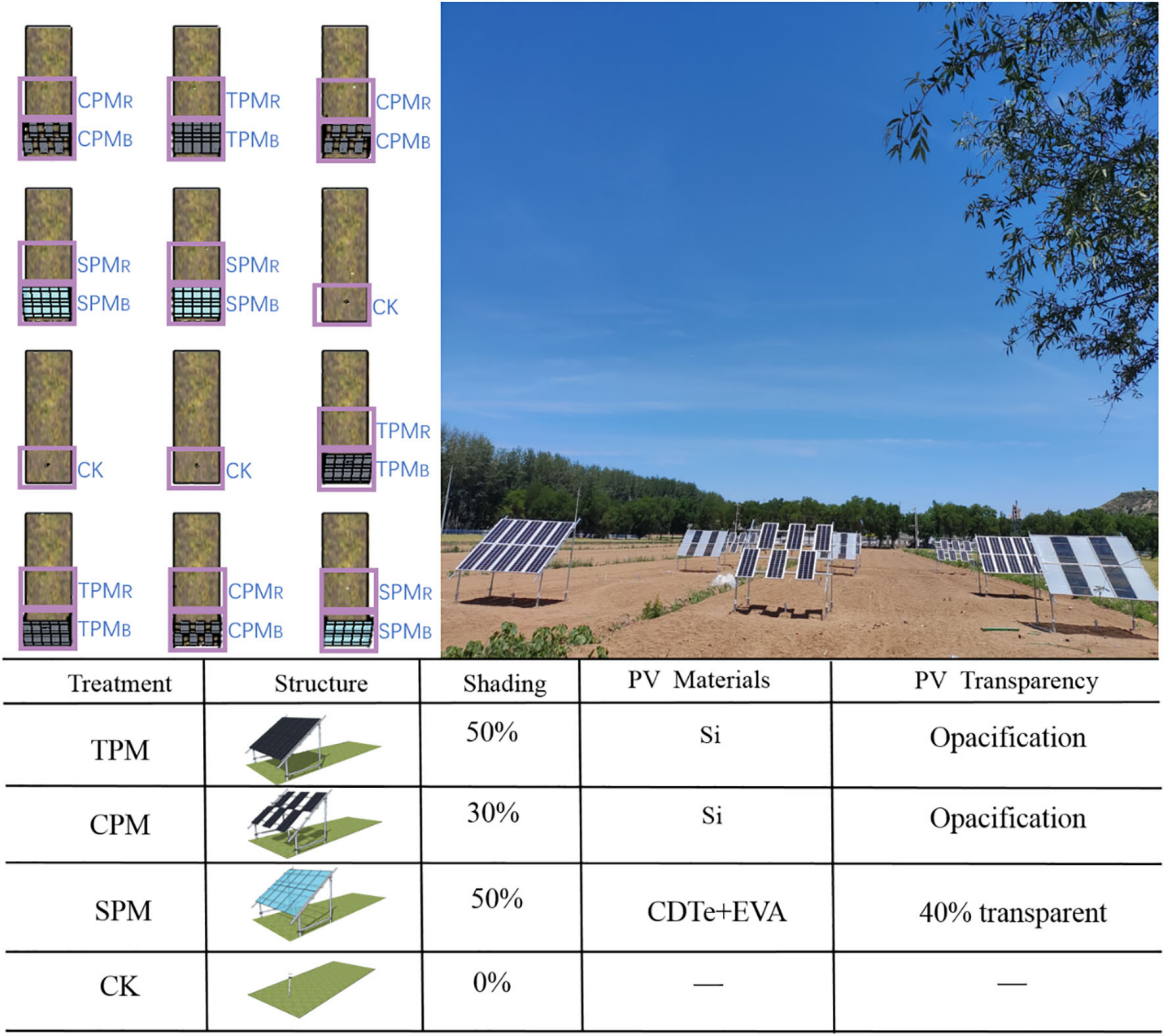
Agrivoltaics cell distribution. Among them, TPM stands for traditional photovoltaic panel, CPM stands for checkerboard photovoltaic panel, SPM stands for translucent photovoltaic panel, and CK represents control treatment. B represents the bottom of the photovoltaic panel, and R represents the rear of the photovoltaic panel.

The experiment was carried out in Beijing’s Shunyi. The average temperature is 11.5°C per year. In this area, there is an average of 2,750 hours of sunshine every year, and there are roughly 195 days without frost. The average precipitation is about 625 millimeters per year, and the average relative humidity is 50%. Four treatments were used in the experiment: CK (control), SPM (translucent photovoltaic panel), CPM (checkerboard photovoltaic panel), and TPM (traditional photovoltaic panel). Furthermore, the impact of photovoltaic panel position on crop yield was considered, with two positions designated: beneath the panel (B) and rear the panel (R). For each of the aforementioned treatments, three replicate cells were established. As demonstrated in **Figure 1**, each constituent element of the photovoltaic system is affixed by four ground stakes, four columns and four crossbars. Photovoltaic panels are positioned above the crossbars and connected to the lines for power generation. The tilt angle of the modules was set at 36°, optimized for maximum solar energy capture based on Beijing’s latitude. The front column measures 80 cm in length, which is the minimum height stipulated to ensure optimal growth conditions for soybeans. This height is also the most commonly employed for ground-mounted power stations.

### 2.2 Crop and field management

**Figure 2:**
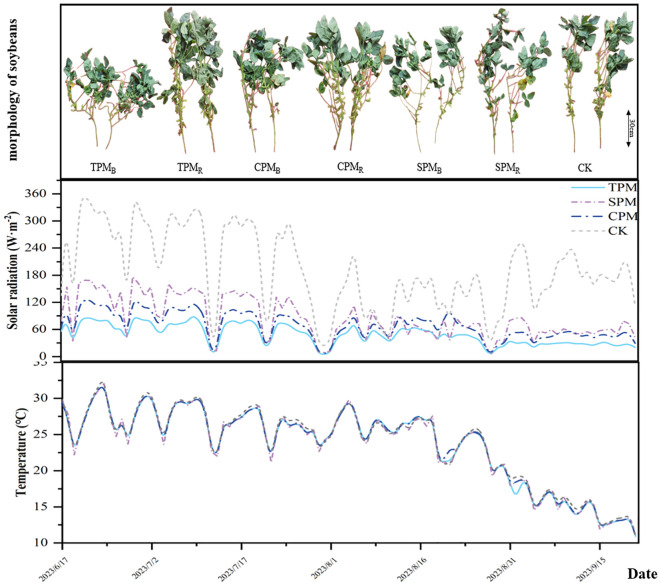
Meteorological conditions and the growth status of crops during the grain filling stage.

The soybean cultivar Zhonghuang 13 was used in this study. Before sowing, 750 kg/km² organic fertilizer was applied (total nitrogen concentration ≥12%, organic matter concentration ≥30%), and the nutrient status of the field soil was determined (active phosphorus 21.66 mg·kg^−1^, available potassium 103.6 mg·kg^−1^, hydrolyzed nitrogen 89.44 mg·kg^−1^). Soybeans were sown on June 22, 2023, with a row spacing of 40 cm and a plant spacing of 10 cm. The grain-filling stage commenced on 31 August 2023, at which point the first sampling was conducted. Subsequent samplings were performed at 11, 20, and 41 days after the onset of grain filling (four samplings total), with final harvest occurring after the last sampling. **Figure 2** illustrates soybean growth dynamics during the grain-filling stage alongside corresponding meteorological data. Other treatments such as weeding (herbicide was applied after seeding and during flowering), and insecticide application (insecticide was applied during flowering) were consistent with local farming practices.

### 2.3 Detection and processing

#### 2.3.1 Net photosynthetic rate

Net photosynthetic rate (Pn) measurements were conducted between 9:00 and 11:00 AM on clear, windless days with minimal cloud cover. For each experimental plot, six healthy plants with uniform leaf development and no visible signs of disease or pest damage were selected. The net photosynthetic rate was measured on functional leaves (middle leaflets of the second fully expanded trifoliate leaves from the apex) using a portable photosynthesis system (LI-6400XT, LI-COR Biosciences, USA). The monitoring was carried out once at the branch stage, flowering stage, pod stage and maturity stage of soybean, while the grain filling stage was monitored three times.

#### 2.3.2 Nitrogen concentration

In this experiment, four times of samples were collected from the grain filling stage to the maturity stage, with four soybean plants randomly selected from each plot during the grain filling stage. The soybean seeds were separated from other organs (stems, leaves, and pods). Samples were oven-dried at 105°C for 30 min, followed by drying at 80°C to constant weight. Dried samples were weighed and ground (< 0.5 mm) for subsequent analysis. Total nitrogen concentration in soybean grains and other organs was determined by Kjeldahl method. The formula for calculating nitrogen accumulation in soybean is as follows (Equations 1, 2):


(1) $$N_{g}=C_{g}\times W_{g}$$

(2) $$N_{t}=C_{g}\times W_{g}+C_{o}\times W_{o}$$

$N_{g}$ represents the grain nitrogen accumulation, $C_{g}$ represents the grain nitrogen concentration, and $W_{g}$ represents the grain dry matter weight. $N_{t}$ represents total nitrogen accumulation, $C_{o}$ represents nitrogen concentration of other organs, and $W_{o}$ represents dry matter weight of other organs.

#### 2.3.3 Crude protein concentration

Crude protein concentration was determined using near-infrared spectroscopy (NIRS). For each plot, harvested soybean samples were homogenized and placed in the analyzer’s sample cell. Each sample underwent duplicate measurements following standard NIRS protocols: after initial analysis, samples were thoroughly remixed before the second measurement to ensure representative sampling. The mean value of the two measurements was recorded as the plot’s crude protein concentration.

#### 2.3.4 Model fitting and processing

In this study, the logistic model was used to simulate the grain nitrogen concentration of soybean at the grain filling stage. Since the earliest detection of grain nitrogen accumulation can only start from the grain filling stage, the simulation period of grain nitrogen accumulation in this study was from the grain filling stage to the maturity stage. The specific formula (Yao et al., 2011) is as follows (Equations 3, 4):


(3) $$y=k/exp\left(r\times \left(t-t_{0}\right)\right)$$

(4) $$I_{max}=\frac{k}{4}\times r$$

Where t are the constant parameters of the model. Among them, k represents the maximum nitrogen accumulation potential, which is the potential of soybean grain nitrogen to reach nitrogen accumulation. r stands for primary growth rate, which is the ability of soybean grains to absorb nitrogen in the early stage. t represents the time it takes to reach the maximum growth rate; $I_{max}$ represents the maximum nitrogen absorption efficiency of soybean grains and the maximum capacity of soybean grains to absorb nutrients.

#### 2.3.5 Path analysis

Path analysis has been shown to provide a more flexible modeling approach in situations characterized by complex multicollinearity issues. The path analysis in this study was conducted using SPSSAU. During the model construction process, the leaf and stem indicators of above-ground parts were initially defined as exogenous variables, the parameters of the logistic model as mediating variables, and the crude protein concentration of soybean grains as the endogenous variable. However, this initial specification failed to meet the required model fit criteria. Consequently, the model was revised by allowing the indicators prior to the grain filling stage to directly influence the crude protein concentration of soybean grains. After a series of iterative modifications, the model achieved an acceptable level of fit: the chi-square to degrees of freedom ratio (χ²/df) was below the commonly accepted threshold of 5, P > 0.05, and multiple fit indices—including the Normed Fit Index (NFI), Relative Fit Index (RFI), Incremental Fit Index (IFI), Comparative Fit Index (CFI), and Tucker-Lewis Index (TLI)—all exceeded the recommended threshold of 0.90.

### 2.4 Data analysis

The primary objective of this study is to elucidate the impact pathway of photovoltaic agriculture on nitrogen dynamics in soybean. To achieve this, one-way ANOVA was conducted to analyze nitrogen concentration, nitrogen accumulation, and crude protein concentration in soybean. The pattern of grain nitrogen accumulation was modeled using the least squares method, and the associated nitrogen accumulation parameters were further examined through two-factor ANOVA. Finally, path analysis was employed to quantify the effects of photovoltaic agriculture on the crude protein concentration of soybean. All data analyses were performed using SPSS and SPSSAU software.

## 3 Results

### 3.1 Net photosynthetic rate and nitrogen transfer in leaves and stems

**Figure 3:**
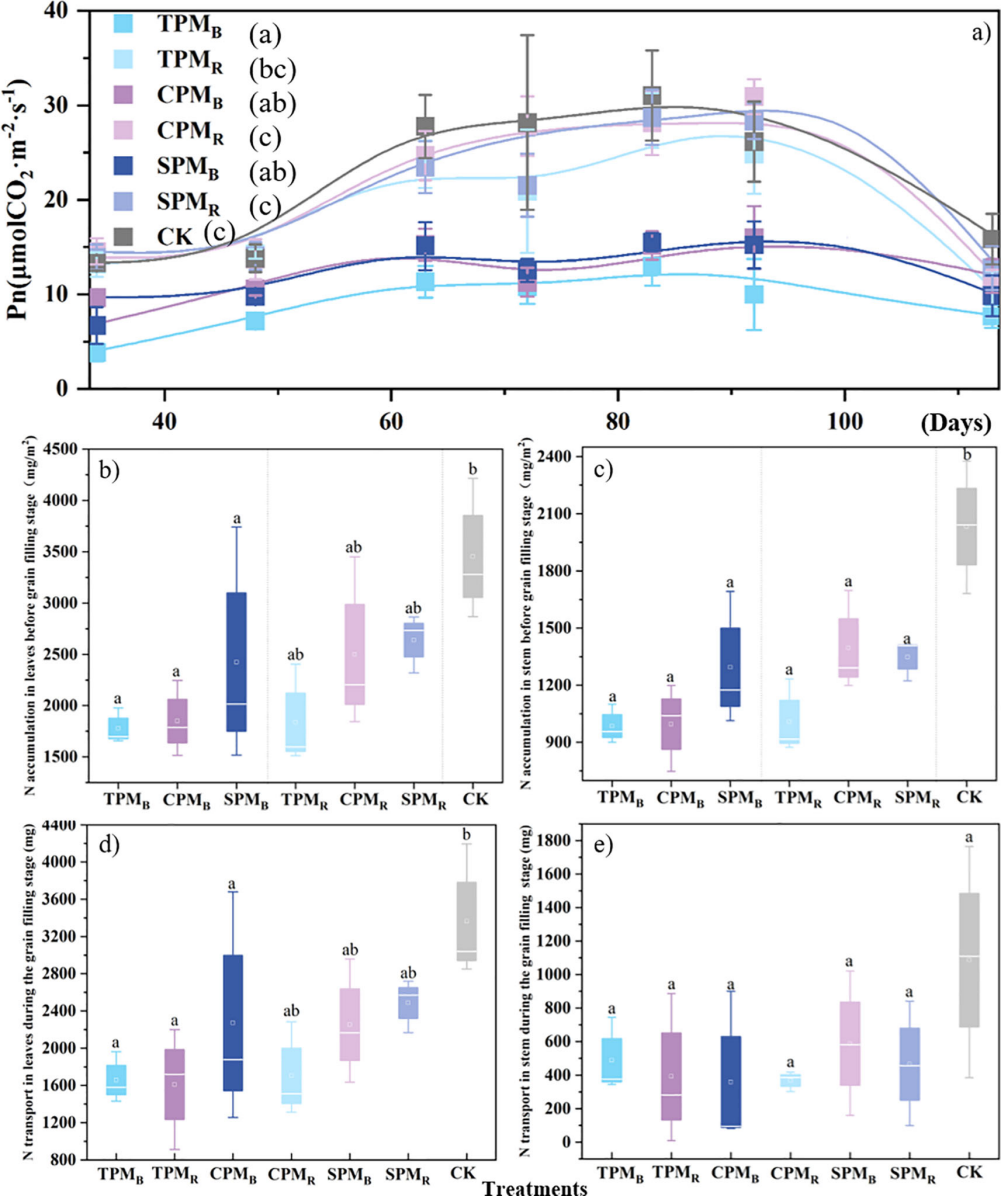
Photosynthetic rate, nitrogen accumulation, and transfer in leaves and stems. In **(a–e)**, TPM, CPM, SPM, and CK represent an ordinary photovoltaic panel, a checkerboard photovoltaic panel, a translucent photovoltaic panel (with a light transmittance of 40%), and a comparison with field treatment, respectively. B and R denote the positions directly below and rear of the photovoltaic panel, respectively. Different lowercase letters represent significant differences between treatments at the 5% level. In **(a)**, the number represents the number of days entering the grain filling stage.

As demonstrated in **Figure 3a**, the position of the agrivoltaics treatments exerted a substantial influence on the net photosynthetic rate of soybean. The net photosynthetic rates of the TPM_B_, CPM_B_, and SPM_B_ treatments, located directly below the photovoltaic panel, exhibited significant decreases of 59.10%, 44.84%, and 43.32%, respectively, compared to the CK treatment. As demonstrated in **Figures 3b, d**, the leaf nitrogen accumulation and transfer volume of TPM_B_, CPM_B_, and SPM_B_ were significantly reduced in comparison with the CK treatment. As demonstrated in **Figures 3c, e**, the stem nitrogen accumulation and transfer volume under agrivoltaics treatment were found to be significantly lower compared to the CK treatment.

### 3.2 Crude protein concentration and nitrogen accumulation in soybean grains

**Figure 4:**
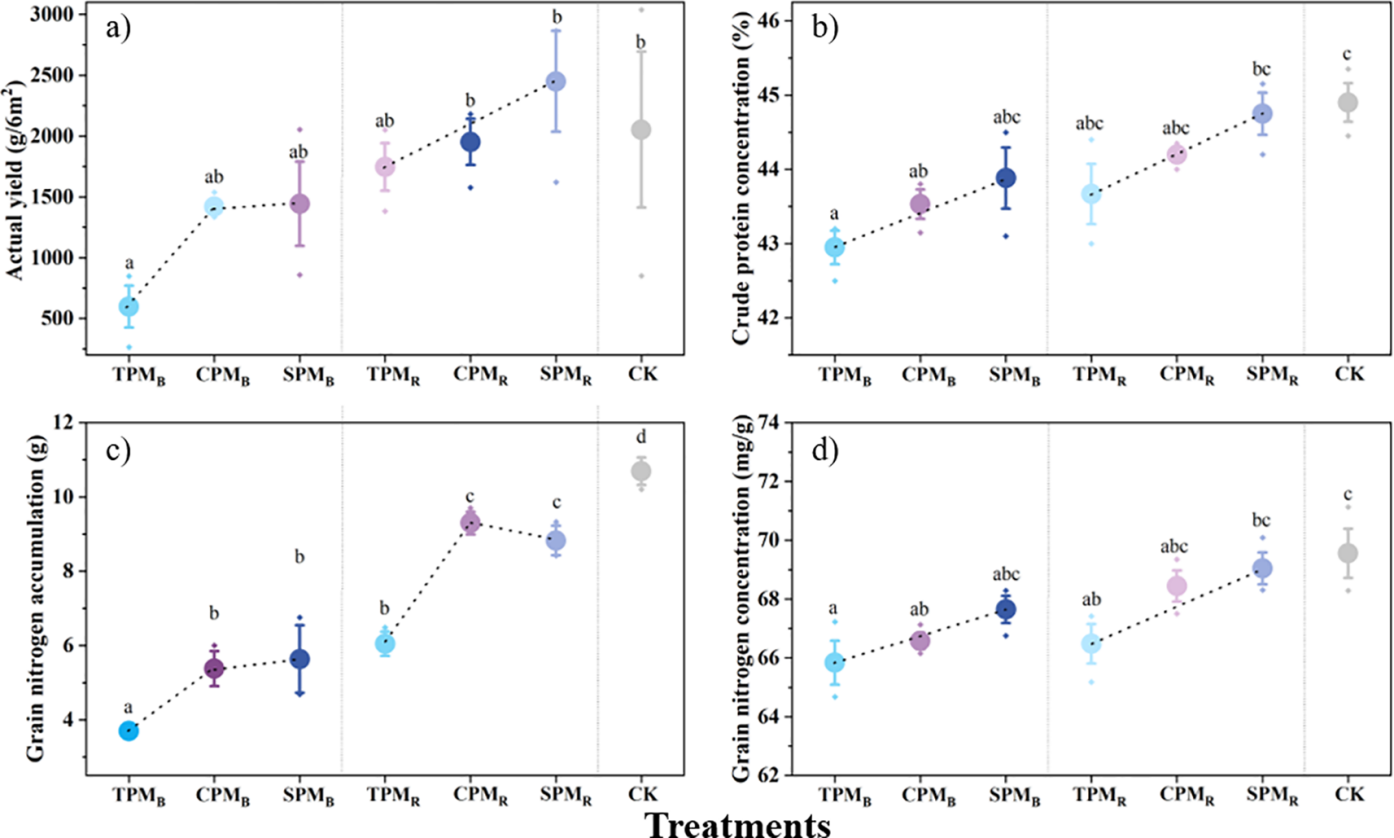
Grain yield and protein levels between treatments. In **(a-d)**, TPM, CPM, SPM, and CK represent an ordinary photovoltaic panel, a checkerboard photovoltaic panel, a translucent photovoltaic panel (with a light transmittance of 40%), and a comparison with field treatment, respectively. B and R denote the positions directly below and rear of the photovoltaic panel, respectively. Different lowercase letters represent significant differences between treatments at the 5% level.

The translucent PV panels demonstrated superior performance in terms of yield and quality concentration in comparison to alternative PV treatments. As demonstrated in **Figure 4a**, regarding yield, the TPMB treatment yielded a significantly lower result compared to the control treatment. The results for crude protein concentration and grain nitrogen concentration demonstrated no significant difference between soybeans treated directly below and rear the translucent PV panels and the control treatment (**Figures 4b, d**). In contrast, all PV treatments were significantly lower than the control treatment in terms of seed nitrogen accumulation (**Figure 4c**).

**Figure 5:**
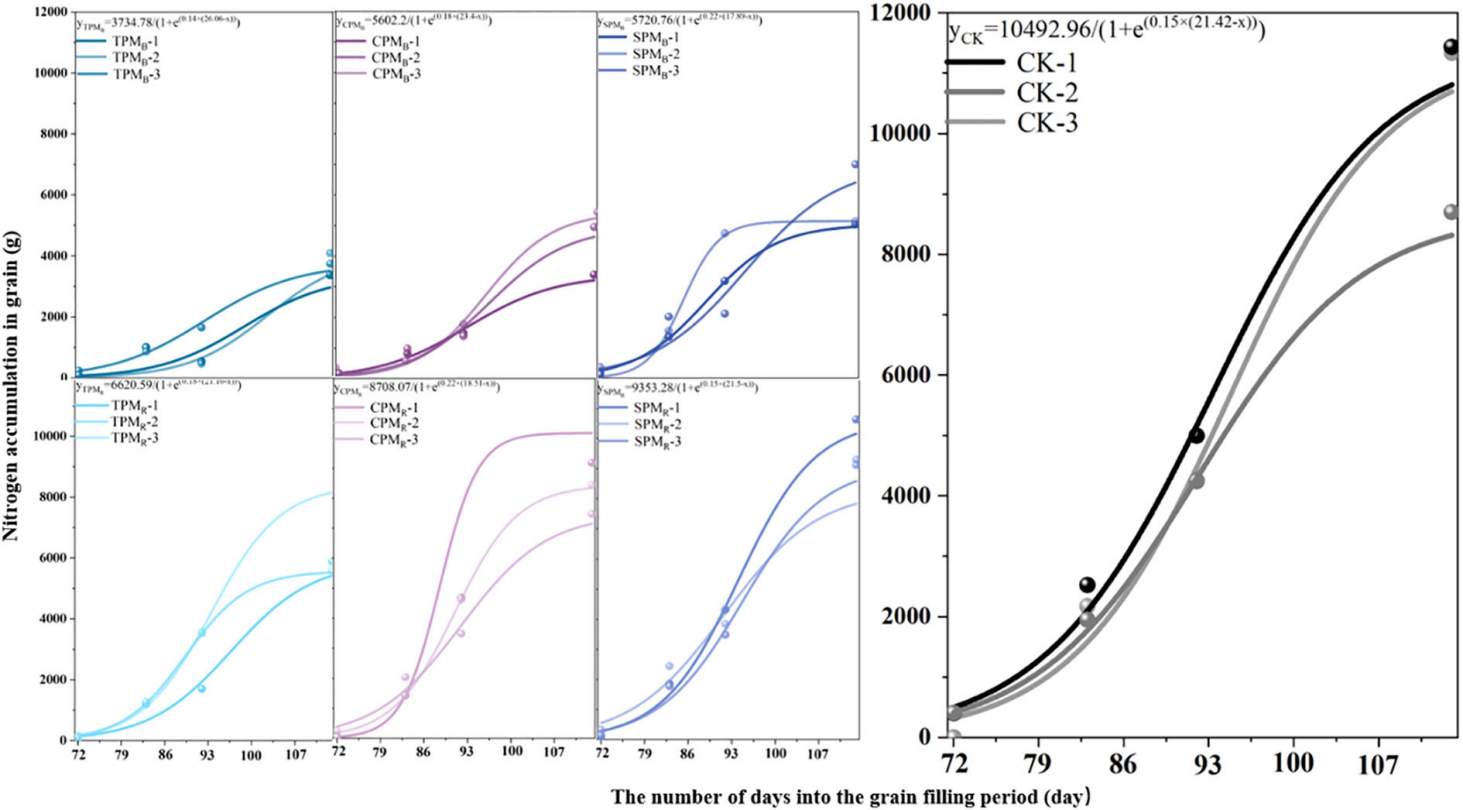
Grain nitrogen accumulation and its parameters. TPM, CPM, SPM, and CK represent an ordinary photovoltaic panel, a checkerboard photovoltaic panel, a translucent photovoltaic panel (with a light transmittance of 40%), and a comparison with field treatment, respectively. B and R represent the positions directly below and rear of the photovoltaic panel, respectively. The use of lowercase letters in **Figures 2a, b** denotes statistical significance at the 5% level among the various treatments. In **Figure 2c**, grain-1, grain-2, and grain-3 represent the three replicates of the same treatment, respectively. The equations were obtained by logistic simulation.

As illustrated in **Figure 5**, the growth dynamics of soybean grain nitrogen during the grain-filling stage exhibited consistency with the logistic model, and the simulated correlation coefficients were all greater than 0.87. As demonstrated in **Table 1**, the treatments, locations, and interaction of photovoltaic modules have substantial effects on the parameters of soybean grain accumulation. The maximum nitrogen accumulation potential (k) of grain exhibited a substantial response to treatments and locations. A comparison of treatments revealed that the k value of grain nitrogen accumulation under CPM and SPM treatment was significantly higher than that under TPM treatment. Moreover, the k value of grain nitrogen accumulation in rear photovoltaic treatment was found to be significantly higher than that observed in the below photovoltaic treatment. The maximum nitrogen absorption efficiency (Imax) reflected the maximum rate of grain nitrogen absorption, which was primarily influenced by the location of photovoltaic treatment. The Imax values of TPM_R_, CPM_R_, and SPM_R_ exhibited a substantial increase of 117.16%, 16.82%, and 92.68%, respectively, compared to TPM_B_, CPM_B_, and SPM_B_. The t value of grain nitrogen accumulation in agrivoltaics is defined as the number of days required to reach the maximum accumulation rate. A substantial t value suggests that the process of nitrogen accumulation is inadequate. The t value of grain nitrogen accumulation under TPM treatment was significantly delayed in comparison with that under SPM treatment.

**Table 1:** Parameters of nitrogen accumulation in soybean seeds under photovoltaic treatment.

Treatment	k	r	t	Imax
TPM_B_	3734.78 ± 297.52aA	0.14 ± 0.01aA	26.06 ± 4.24bA	134.29 ± 16.68aA
CPM_B_	5602.2 ± 2110.14bA	0.18 ± 0.04aA	23.4 ± 1.98abA	270.38 ± 162.2aA
SPM_B_	5720.76 ± 903.44bA	0.22 ± 0.09aA	17.89 ± 4.09aA	300.01 ± 103.32aA
TPM_R_	6620.59 ± 1261.96aB	0.18 ± 0.02aA	21.16 ± 3.01bA	291.63 ± 63.6aB
CPM_R_	8708.07 ± 1114.23bB	0.22 ± 0.08aA	18.51 ± 1.43abA	520.98 ± 220.39aB
SPM_R_	9353.28 ± 963.75bB	0.15 ± 0.01aA	21.5 ± 1.25aA	350.48 ± 63.69aB
CK	10492.96 ± 1269.94cC	0.15 ± 0aA	21.42 ± 1.06abA	391.47 ± 49.6aC

Among them, “a”, “b” and “c” represent the difference between photovoltaic panel processing; “A”, “B” and “C” represent the difference between locations; Treatment has a significant impact on k value, location has a significant impact on Imax value, and processing × location has a significant impact on t value.

### 3.3 Analysis of the influence path of photovoltaic treatment on crude protein concentration

**Figure 6:**
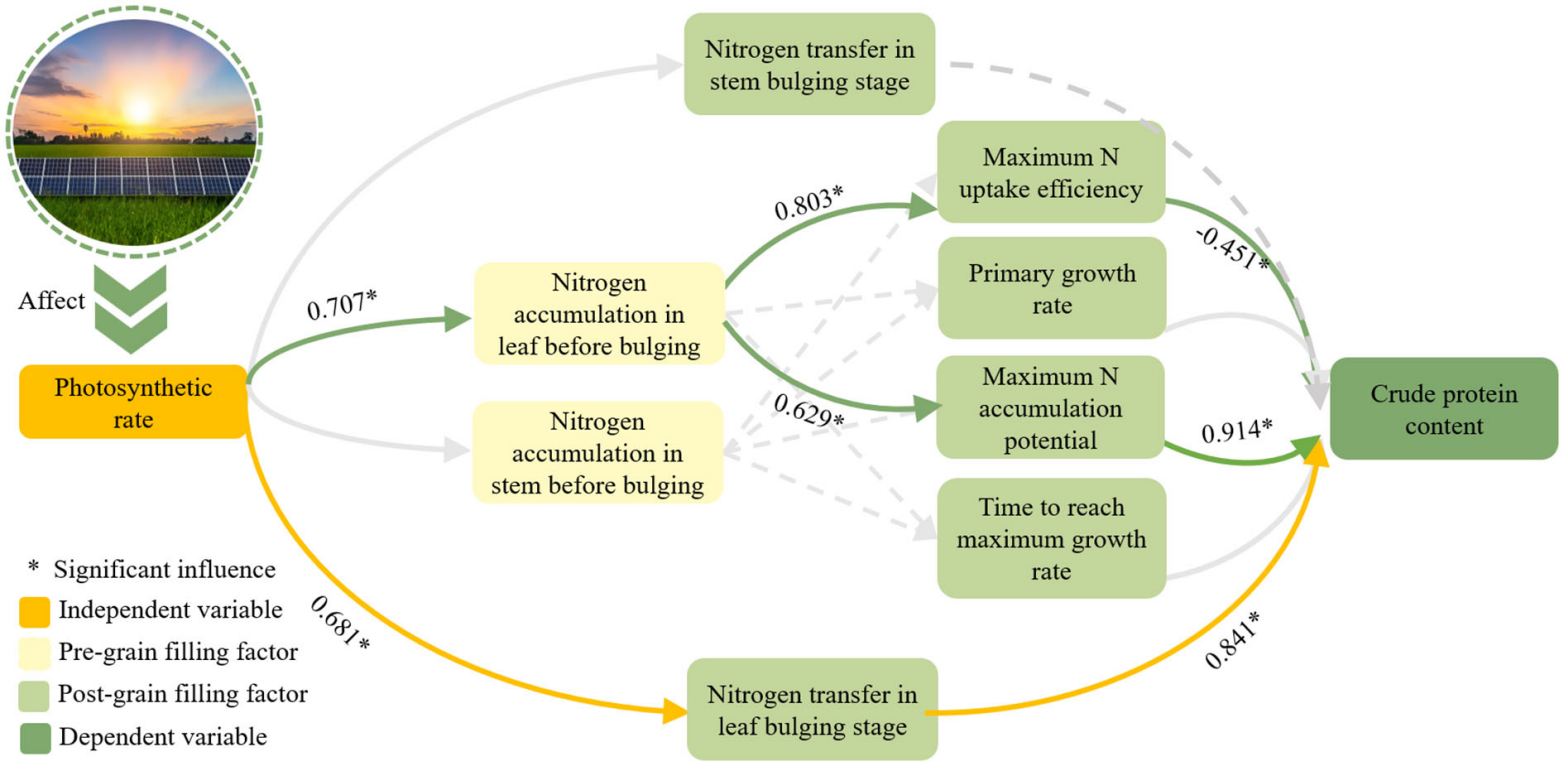
The influence path of grain crude protein concentration.

Following the implementation of agrivoltaics, a shift in the photosynthetic activity of plants was observed, which affected the nitrogen accumulation in grains. Gaju et al. (2014) proposed that grain nitrogen accumulation was affected by two paths of nitrogen re-mobilization after flowering and nitrogen accumulation before flowering. The present experiment demonstrated that the specific influence path of photovoltaic panel erection on soybean quality (**Figure 6**) was as follows: (1) Crude protein concentration was found to be influenced by the amount of leaf nitrogen transferred during the grain-filling stage. (2) The accumulation of nitrogen in leaves before the filling period affects the nitrogen absorption process of grains, and thereby influences the concentration of crude protein. The course of leaf nitrogen accumulation prior to the grain filling stage exhibits two divergent trajectories in grain nitrogen uptake. On the one hand, it exerts a positive influence on grain crude protein concentration by modulating the maximum potential of grain nitrogen accumulation. On the other hand, it hurts the grain crude protein concentration by influencing the maximum nitrogen accumulation efficiency. In contrast, the amount of stem nitrogen accumulation prior to the grain filling stage, as well as the amount of stem nitrogen transfer during the grain filling stage, exhibited no significant impact on the grain crude protein concentration.

The net photosynthetic rate had a substantial positive impact on leaf nitrogen accumulation and transfer prior to the grain filling stage. The influencing factors were determined to be 0.707 and 0.681, respectively. Through path analysis, Path 1 was identified as the effect of leaf nitrogen accumulation on promoting grain nitrogen accumulation prior to the grain filling stage. This ultimately affected the crude protein of the grain. The effects of k value and Imax on crude protein concentration were found to be 0.914 and -0.451, respectively. Path 2 was that the nitrogen transfer of leaves would directly affect the crude protein concentration positively, with the influencing factor reaching 0.841. In general, the maximum nitrogen accumulation potential and the amount of nitrogen accumulated by leaves before the grain-filling stage had significant positive effects on crude protein. They were the key factors affecting the production of soybean protein.

Among them, the independent variable refers to the primary variable that affects the installation of agrivoltaics; the pre-grain filling factor refers to the factors that change in response to the independent variable before the soybean enters the grain filling stage. Post-grain filling factor, conversely, refers to the factors that change with the independent variable or pre-grain filling factor after the soybean enters the grain filling stage. The dependent variable is defined as the variable that is influenced by the aforementioned three variables. In this study, the primary focus is on the concentration of crude protein.

## 4 Discussion

In this study, soybean cultivation under photovoltaic modules showed no significant difference in leaf nitrogen accumulation between the SPM_R_ treatment and the control. However, the TPM_B_, CPM_B_, and SPM_B_ treatments exhibited a marked decline in leaf nitrogen concentration (**Figure 3**). These findings are consistent with previous studies on shading, which have reported that altered phytohormone levels can affect leaf development (Oritani, 1978; Hu et al., 2021), ultimately leading to reduced leaf nitrogen concentration and premature leaf abscission (Pons and Pearcy, 1994). Notably, translucent PV panels appear to mitigate nitrogen depletion in vegetative tissues, resulting in higher foliar nitrogen concentrations compared to traditional PV systems (Potenza et al., 2022b; Thompson et al., 2020). Since leaf and stem nitrogen dynamics directly influence soybean quality, these results underscore the importance of PV panel selection in agrivoltaic systems.

As shown in **Figure 4**, the SPM_B_ and SPM_R_ treatments did not significantly reduce crude protein concentration or nitrogen accumulation in soybeans. In contrast, other agrivoltaic treatments led to a decline in crude protein concentration. Specifically, the TPM_B_ and CPM_B_ treatments exhibited significant reductions in crude protein concentration by 4.34% and 3.04%, respectively, compared to the control. Similarly, nitrogen accumulation decreased substantially by 5.35% and 4.28% in these treatments. This effect may be linked to shading-induced inhibition of nitrogen translocation to grains, ultimately compromising soybean protein quality (Asanome and Ikeda, 2000). However, recent studies by Lu et al. (2024) and Zaky et al. (2024) suggest that while grain protein concentration may decrease under certain agrivoltaic conditions, strategic panel placement (e.g., behind photovoltaic modules) or the use of translucent panels can enhance light availability for crops, thereby maintaining grain quality.

Furthermore, we employed a Logistic model to analyze nitrogen accumulation dynamics in grains across different treatments. The SPM_R_ treatment demonstrated the highest maximum nitrogen accumulation potential, reaching 9353.28 ± 963.75 mg (**Table 1**), consistent with findings by Li et al. (2008) and Pan et al. (2006). They found that lower reductions in solar radiation intensity corresponded to smaller decreases in grain nitrogen accumulation. Furthermore, the difference in treatment duration between SPM_R_ and CK was merely 0.08 days. Previous studies have indicated that premature attainment of the maximum nitrogen accumulation rate in grains can shorten the crop growth period (Zhang et al., 2015), while a delayed reduction in the average nitrogen accumulation rate can also affect crop development (Giunta and Motzo, 2005). These results collectively indicate that SPM_R_ exhibits superior nitrogen capture efficiency and demonstrates the best stability during nitrogen accumulation processes.

As illustrated in **Figure 6**, the Logistic model revealed a substantial impact of grain nitrogen accumulation parameters on crude protein concentration. Notably, the maximum grain nitrogen accumulation rate exhibited a significant negative correlation with crude protein concentration (-0.451). Pan et al. (2006) and Fabre and Planchon (2000) posited that the maximum rate of nitrogen accumulation exhibited a positive correlation with crude protein concentration. This is because Pan et al. regarded the rate of nitrogen accumulation as a constant value; thus, an elevated rate of nitrogen accumulation corresponded to an enhanced quality. In this study, the nitrogen accumulation rate was dynamic. The larger the maximum nitrogen accumulation rate is, the narrower the integral area consisting of the rate will be. This phenomenon resulted in a reduction in both grain nitrogen accumulation and crude protein concentration (Imsande, 1992; Nehe et al., 2020). A significant positive correlation was identified between maximum nitrogen accumulation potential and crude protein concentration (r = 0.914). One potential explanation for this phenomenon is that the source restriction of reduced photosynthesis hindered the transport of nitrogen from vegetative organs to grains (Gao et al., 2020), thereby reducing the potential for nitrogen accumulation in grains and consequently affecting the quality of soybeans (Makino, 2011). The leaf nitrogen transfer during the grain-filling stage exhibited a significant positive correlation with crude protein concentration (r = 0.841). In contrast, no significant correlation was observed between stem nitrogen transfer and crude protein concentration (**Figure 6**). This phenomenon can be attributed to the fact that leaves and roots serve as primary nitrogen sinks during the vegetative development stage (Masclaux-Daubresse et al., 2010) and therefore contain higher levels of nitrogen. During the grain filling stage, nitrogen from the leaves is translocated to the grains (Mikesell and Paulsen, 1971), thereby limiting grain protein synthesis due to the depletion of the leaf nitrogen pool (Barneix and Guitman, 1993). Consequently, to prioritize the expansion of leaf area, the allocation of nitrogen to stems and other structures is decreased (Gastal and Lemaire, 2002). Therefore, nitrogen transfer in the stem was lower than that in the leaf, and was not strongly correlated with crude protein concentration.

Leaf nitrogen accumulation prior to the grain-filling stage, the amount of leaf nitrogen transferred, and grain nitrogen accumulation potential during the grain-filling stage had significant positive effects on crude protein concentration. Sarakhsi et al. (2010) suggested that the application of foliar fertilizers could enhance leaf nitrogen accumulation. Furthermore, Zakeri et al. (2015) proposed that utilizing available nitrogen during the grain nitrogen absorption process could promote nitrogen accumulation in grains, thereby increasing the potential for nitrogen accumulation. Based on the existing literature, it can be inferred that under agrivoltaics installation conditions, the judicious application of foliar fertilizer prior to the grain filling stage and the utilization of available nitrogen during this stage may mitigate the adverse effects of agrivoltaics installations on soybean quality.

## 5 Conclusion

This study elucidates the effects of agrivoltaics systems on crop quality and nutrient accumulation processes in the field. Through experiments, Experimental results reveal a continuous and quantitative pathway of agrivoltaics affecting crude protein concentration. The reduction in solar irradiance within agrivoltaics systems impacted leaf nitrogen accumulation and transfer prior to the grain filling stage, consequently decreased in crude protein concentration. These findings enhance our understanding of grain quality variations under agrivoltaic conditions. During the implementation of agrivoltaic systems, foliar fertilizer application can enhance leaf nitrogen accumulation, thereby mitigating potential adverse effects on soybean quality. Moreover, the treatment involving translucent photovoltaic panels installed at the rear position exhibited the maximum nitrogen accumulation rate and total nitrogen accumulation most similar to those of the control treatment. The influence of solar radiation intensity on crude protein concentration became negligible in this configuration, highlighting the potential application of translucent photovoltaic panels in agrivoltaic systems. Under field conditions, these panels can help preserve soybean protein quality and provide innovative strategies for climate change adaptation.

## Data Availability

The data analyzed in this study is subject to the following licenses/restrictions: Readers can request the data by contacting the author. Requests to access these datasets should be directed to 82101222003@caas.cn.
